# CXC chemokine ligand 13 and galectin-9 plasma levels collaboratively provide prediction of disease activity and progression-free survival in chronic lymphocytic leukemia

**DOI:** 10.1007/s00277-023-05540-8

**Published:** 2023-11-09

**Authors:** Heba A. Ahmed, Asmaa Nafady, Eman H. Ahmed, Emad Eldin Nabil Hassan, Walaa Gamal Mohamed Soliman, Mahmoud I. Elbadry, Ahmed Ahmed Allam

**Affiliations:** 1 Department of Clinical Pathology, Faculty of Medicine, Sohag University, Sohag, 82524 Egypt; 2 Department of Clinical and Chemical Pathology, Faculty of Medicine, South Valley University, Qena, Egypt; 3 Department of Clinical Pathology, South Egypt Cancer Institute, Assiut University, Assiut, Egypt; 4 Department of Clinical Oncology and Nuclear Medicine, Sohag University Hospital, Sohag, Egypt; 5 Division of Haematology, Department of Internal Medicine, Faculty of Medicine, Sohag University, Sohag, 82524 Egypt

**Keywords:** Chronic lymphocytic leukemia, CXCL13, Galectin-9, Tumor microenvironment, Anti-tumor immune responses

## Abstract

The clinical outcome of lymphocytic leukemia (CLL) is quite heterogeneous. The purpose of this observational study was to investigate the clinical merit of measuring plasma galectin-9 and CXCL-13 concentrations as predictors of CLL activity, prognosis, and early indicators of therapeutic response. These biomarkers were compared with other prognostic indicators, progression-free survival (PFS), time to first treatment (TTT), and overall survival (OS) over a follow-up period (4 years). First, plasma galectin-9 and CXCL-13 concentrations were analyzed in CLL patients at the time of diagnosis as well as healthy controls. Compared to controls, CLL patients had significantly higher serum levels of CXCL-13 and galectin-9. Second, we observed that CLL patients with high soluble CXCL-13 and galectin-9 levels had advanced clinical stages, poor prognosis, 17p del, short PFS, short TTT, and therapy resistance. The levels of CXCL-13, β2-microglobulin, LDH, CD38%, and high grade of Rai-stage were all strongly correlated with the galectin-9 levels. Soluble CXCL-13 and galectin-9 had very good specificity and sensitivity in detecting CLL disease progression and high-risk patients with the superiority of galectin-9 over CXCL-13. Although the two biomarkers were equal in prediction of TTT and treatment response, the soluble CXCL13 was superior in prediction of OS. High CXCL-13 and galectin-9 plasma levels upon CLL diagnosis are associated with disease activity, progression, advanced clinical stages, short periods of PFS, short TTT, and unfavorable treatment response.

**Supplementary Information:**

The online version contains supplementary material available at 10.1007/s00277-023-05540-8.

## Introduction

Chronic lymphocytic leukemia (CLL), the most frequent type of leukemia in adults, is a lymphoproliferative disorder that is characterized by the expansion of monoclonal, mature CD5 + CD23 + B cells in the peripheral blood, secondary lymphoid tissues, and bone marrow [1].

A high incidence of heterogeneity in the clinical outcomes was observed among CLL patients, with some patients surviving for many years without any therapy and eventually succumbing to unrelated diseases, whereas others die rapidly, within 2–3 years of diagnosis, due to CLL complications despite receiving aggressive chemoimmuno or targeted therapy [2, 3]. Therefore, it is essential to accurately assess the prognosis of CLL patients.

Individual, specific cytokines and chemokines have been reported to be elevated in the sera, plasma, or both of CLL patients and correlate with clinical course and outcome [4]. The immunoglobulin heavy-chain variable region (IGHV) mutational status, which predicts clinical outcome at diagnosis, is presently the most important prognostic marker in CLL [5]. Other markers, including as CD38, deletion of 17p (17p del), and ZAP70, have also been studied as IGHV surrogates [6, 7]. In untreated CLL patients, these markers often remain steady over time. Therefore, there is a requirement for markers that can track specific patients throughout the course of the disease, and additional precise prognostic markers are required to continue improving patient outcomes. Also, routine detection of these biomarkers, on the other hand, is not a cost-effective test and is hence not commonly used.

The B cell-attracting chemokine CXC ligand 13 (CXCL-13) identified as a critical homeostatic chemokine that is expressed in associated lymphoid tissues, and B cells and follicular T helper (TFH) cells [8], whereas galectin-9 (Gal-9) is a member of the galectin family that is constitutively expressed in antigen-presenting cells and induced by interferon β in cancer cells [9]. CXCL-13 and galectin-9 are among the key chemotactic factors which play crucial roles in malignancy and lymphoid malignancies [10–14]. Recent reports have shown the significance of CXCL-13 in CLL disease activity [15, 16] that could be useful prognostic markers for CLL. Also, previous research found that serum galectin-9 is significantly higher in CLL patients as well as oral cancer, pancreatic cancer, and other hematologic malignancies [12–14]. Taghiloo et al. [17] discovered that galectin-9 levels were 30 times higher in the malignant cells of CLL patients which resulted in T cell exhaustion and an imbalance of CD4 + T cell subsets in CLL.

However, the prognostic value of galectin-9 and CXCL-13 in CLL pairs has not been fully understood. It is still critical to define the roles of these novel indicators about disease courses that may aid in patient treatment decision-making. Also, no study to date has explored the correlation between galectin-9 and CXCL-13 plasma concentrations in CLL patients or compared their collaborative accuracy as prognostic biomarkers.

Our ultimate objectives are to offer reliable predictors of CLL activity and prognosis as well as early indicators of therapeutic response. Therefore, we investigated the clinical merit of measuring plasma galectin-9 and CXCL-13 concentrations together in CLL in a longitudinal prospective study.

## Methods

### Patients and healthy volunteers

We carried out a longitudinal prospective observational study at Sohag University Hospitals over a 54-month period. Ninety-one newly diagnosed and treatment-naive CLL patients were included in this prospective study. Fifty sex-matched and age-matched healthy volunteers were included as controls. The study protocol was revised and approved by the Medical Research Ethics Committee of Sohag Faculty of Medicine (number: Soh-Med-21–11-32). Before participating in the study and collecting samples, informed consent was obtained from all patients in accordance with the Declaration of Helsinki after proper counseling. The immunophenotypic and clinical criteria for CLL were all met by all patients. The following were the exclusion criteria: other malignancy, acute inflammatory disorders, or a history of kidney, liver, or heart failure. Patient demographics, clinical characteristics, and laboratory data were obtained and analyzed. These included the age, sex, spleen size, Rai clinical stage, absolute lymphocyte count, β2-microglobulin and lactate dehydrogenase (LDH) levels, and other variables. As assessed via flow cytometry, CLL bone marrow infiltration was calculated as the proportion of CLL cells to all cells.

### Laboratory investigations

Blood samples (10 mL) were collected from each subject prior to chemotherapy. Complete blood count was performed on EDTA samples XN-1000 (Sysmex, Kobe, Japan). All enrolled patients were screened by microscopic examination of bone marrow (BM) and peripheral blood smears to determine any abnormalities. Immunophenotyping was carried out using Becton Dickinson FACS Caliber Flow Cytometer equipped with CellQuest software (BD Biosciences, San Diego, CA, USA). Serum LDH level was performed on the Chemistry auto analyzer C501 (La Roche Ltd, Risch-Rotkreuz, Switzerland). Serum β2 microglobulin was performed using Copas 6000 Analyzer (Roche Diagnostics, Indianapolis, IN, USA).

Serum samples from all enrolled subjects were gathered and were put away at − 20 °C until investigation. Serum CXCL13 and serum galectin-9 levels were estimated by enzyme-linked immunosorbent assay (ELISA) (R&D Systems Inc., Minneapolis, MN, USA). Serum CXCL13 and serum galectin-9 levels were evaluated by the manufacturer’s guidelines utilizing a commercially with assay range for CXCL13, 7.8–500 pg/mL and assay range for galectin-9, 0.2–10 ng/mL.

The clonal 17p del was evaluated in 21 CLL patients using fluorescence in situ hybridization (FISH) on interphase nuclei derived from cultured bone marrow cells using a probe designed to detect deletion of 17p13.1 (TP53) according to the manufacturer’s instructions (Abbott Molecular, IL). The total number of cells (200 cells) was counted for FISH probe (Figure S1). See the supplemental material for more information about the diagnosis and many modalities of radiographic and histological studies were used.

### Treatment and follow-up parameters

The International Workshop on Chronic Lymphocytic Leukemia (IWCLL) guidelines were used to determine whether chemotherapy or a “watchful waiting” strategy should be used. Therapy was offered to all patients who need active treatment and met the 2018 IWCLL guidelines [18]. Many available treatment modalities in addition to supportive measurements were given to patients according to schedules included, cyclophosphamide, vincristine, and prednisone (CVP), or fludarabine, cyclophosphamide ± rituximab (FCR), or Bruton tyrosine kinases inhibitors (BTKIs) as ibrutinib depending on the patient’s clinical condition (like patient’s age, comorbidity, and status of 17p13 deletion).

The patients were followed monthly until the time of writing this manuscript. During this period, time to first therapy (TTFT), laboratory profiles, criteria of treatment response including size of lymph nodes, spleen, and liver, and hemoglobin levels, lymphocytes, neutrophils, and platelets numbers were checked. Histopathological studies of BM were performed according to indication. All patients were scanned by ultrasonography and examined by pan computerized tomography (Pan-CT) to view the size of lymph nodes, spleen, and liver size. The significant events were documented, including treatment complication, BM failure, progression-free survival (PFS), and overall survival (OS). The period between the time of follow-up and the occurrence of disease progression or death from any cause was defined as PFS. The term “time to treatment” (TTT) was used to describe the interval between the time of sample collection and the date of therapy. The response to the difference types of treatments was evaluated according to IWCLL criteria [18].

### Statistical analyses

SPSS 20 software was used to conduct statistical analysis (SPSS, Inc., Chicago, IL). To analyze differences between categorical variables such as spleen size, lymphadenopathy, and Rai stage risk were represented as frequency and percentage. Continuous variables, such as age, white blood counts (WBC), and β2-microglobulin levels were represented as median, range, mean, and standard deviation (SD). The Shapiro–Wilk test was used to determine the normality of the variables. The Fisher’s exact test or Pearson’s χ^2^ test was used to evaluate the differences of dichotomous variables. Mann–Whitney *U* test and Kruskal–Wallis were utilized to compare each of the continuous variables between dichotomized categories factors. The receiver operating characteristic (ROC) curve was used to establish the best cut-off point for each type tested CXCL13 and galectin 9 to distinguish between patients and controls, as well as CLL patients’ subtypes. The Spearman rank correlation coefficient was used to examine correlations. ICU and OS of CLL patients in different prognostic groups were assessed using the Kaplan–Meier method. A *p* value of < 0.05 was judged to be statistically significant. GraphPad Prism software package, version 5.02 (San Diego, CA) was used to make the figures.

## Results

### Patients’ baseline clinical characteristics

Our study population consisted of 91 patients diagnosed as CLL for the first time during the period of this study and 50 healthy age- and sex-matched controls. The median age of our patients was 61 (range, 50–80), and 59.3% of them were male. Of the 91 patients, 65 (71.4%) patients had lymphadenopathy and 53 (58.9%) patients had splenomegaly, while 45 (49.5%) had hepatomegaly. CLL patients were classified into three groups based on the Binet staging system: Binet A stage (*n* = 15), Binet B stage (*n* = 55), and Binet C stage (*n* = 21). Also, 21 patients (23.1%) had Rai stage III or IV at diagnosis. Forty-three (47.3%) first presented with anemic manifestation, 31 (34.1%) patients had WBCs ≥ 100 (× 10^3^/µL), and 12 (13%0.2) had thrombocytopenia at diagnosis. There were no significant differences in gender or age between the CLL and healthy groups (all *p* > 0.05). Peripheral blood parameters, on the other hand, differed significantly between the CLL patients and healthy groups (all *p* < 0.001; Table S1).

### Serum CXCL13 and galectin-9 elevated across CLL patients especially high-risk group subtypes

CXCL13 and galectin-9 serum levels were evaluated between a cohort of CLL patients before treatment and healthy controls. We identified that CXCL13 and galectin-9 plasma concentrations were significantly higher in CLL patients, with a median serum CXCL13 concentration of 118.0 pg/mL (range, 40.0–1290.0 pg/mL), and a median galectin-9 concentration of 653.0 pg/mL (range, 300.0–2315.0 pg/mL), compared with CXCL13 concentration of healthy controls (median, 39.5 pg/mL, range, 25.0–250.0 pg/mL) and a median galectin-9 concentration of 274.5 pg/mL (range, 160.0–450.0 pg/mL) (*p* value < 0.0001).

**Fig. 1:**
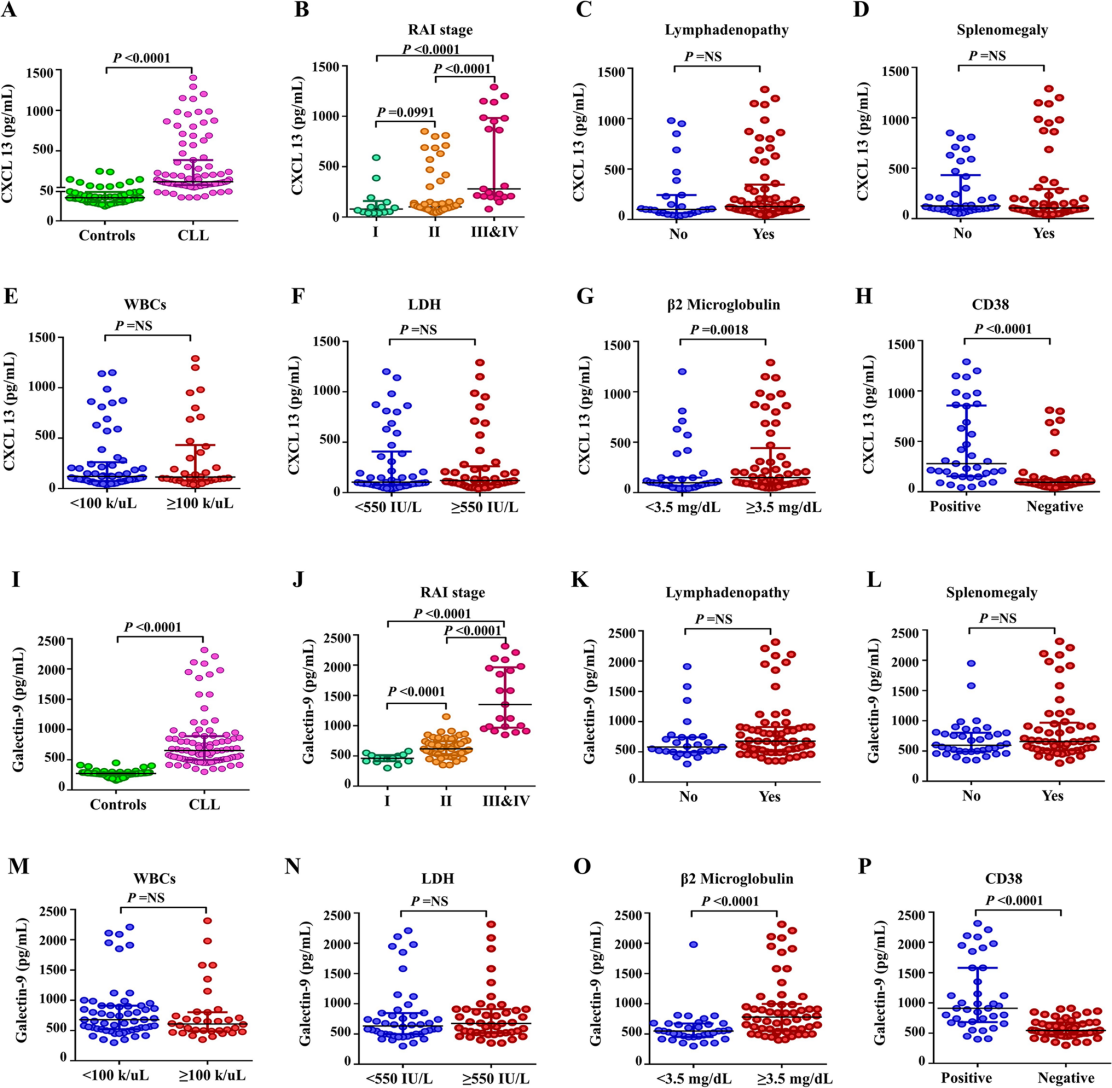
Differential levels of serum CXCL13 and galectin-9 between CLL patients and healthy controls demonstrate increased serum CXCL-13 and galectin-9 across CLL patients especially high-risk group. **A** CXCL13 plasma concentrations in CLL patients and healthy age-matched controls. **B**–**H** Expression of serum CXCL13 in CLL subgroups, i.e., lower- versus higher-risk subsets. **I** Galectin-9 plasma concentrations in CLL patients and healthy age-matched controls. **J**–**P** Expression of serum galectin-9 in CLL subgroups, i.e., lower- versus higher-risk subsets. Horizontal and vertical lines indicate median and interquartile range, respectively; each circle represents an individual patient sample. WBCs white blood counts, LDH lactate dehydrogenase

Second, we evaluated the correlations of CXCL13 and galectin-9 plasma concentrations with CLL activity and disease stages using the established clinical and laboratory markers. Patients with advanced stage of CLL (Rai stage III or IV) had significantly higher CXCL13 plasma levels than early CLL stages (Rai stage I or II) (*p* < 0.0001). Also, galectin-9 plasma levels were significantly higher in patients with advance CLL stages (Rai stage II, III, and IV), compared to Rai stage I patients (*p* < 0.0001). Patients with high plasma concentrations of β2-microglobulin levels (β2M ≥ 3.5 mg/dL) showed a significantly higher median of CXCL13 and galectin-9 plasma concentrations (*p* = 0.0018 and *p* < 0.0001, respectively). Also, patients with positive CD38% expression (> 30%) showed a significantly higher median of CXCL13 and galectin-9 plasma levels (all *p* < 0.0001; Fig. 1).

In the aspect of gender, males had significantly higher galectin-9 plasma concentrations than females (*p* = 0.017). Similar associations were observed in patients with advanced Rai clinical stage who had significantly higher CXCL13 and galectin-9 plasma concentrations (all *p* < 0.0001). Speaking of the association with anemia or thrombocytopenia and CD38 expressions, CLL patients with anemia and/or thrombocytopenia had significantly higher plasma levels of CXCL13 and galectin-9 than patients without these complications (*p* < 0.05, *p* < 0.0001, and *p* < 0.0001, respectively). However, we did not find a significant difference in the plasma levels of CXCL13 and galectin-9 between patients with different clinical phenotypes (elderly, hepatosplenomegaly, lymphadenopathy), higher LDH concentrations, and WBCs count ≥ 100,000/μL. Clonal 17p del studies in 21 cases showed significantly higher CXCL13 and galectin-9 plasma levels in CLL patients with positive 17p del compared to the negative 17p del patients (*p* < 0.002 and *p* < 0.003, respectively) (Table 1).

**Table 1:** Distribution of CXCL-13 and galectin-9 mean/median levels among prognostic factors in 91 CLL patients’ samples

Characteristic	Category	Number	CXCL-13 (pg/mL) Mean ± SD/median (range)	Galectin-9 (pg/mL) Mean ± SD/median (range)	P1 value	P2 value
Age (years)	< 65	68	286.7 ± 330.1/126 (40–1290)	798.3 ± 477.7/635 (300–2315)	0.924	0.753
	≥ 65	23	251.3 ± 310.5/106 (50–980)	787.0 ± 426.5/680 (350–2210)		
Gender	Female	37	234.8 ± 289.1/112 (42–1290)	648.6 ± 326.2/583 (300–2315)	0.519	0.017
	Male	54	307.1 ± 345.3/142 (40–1200)	896.0 ± 516.1/768 (350–2210)		
Liver size	Normal	46	283.9 ± 311.8/131 (40–1290)	833.4 ± 473.6/700 (300–2315)	0.385	0.721
	Mild hepatomegaly	31	255.9 ± 334.7/103 (40–1150)	747.2 ± 414.2/580 (350–2110)		
	Moderate hepatomegaly	14	305.6 ± 360.8/119 (50–1200)	777.6 ± 548.3/520 (415–1980)		
Spleen size	Normal	38	264.2 ± 252.2/98 (50–850)	687.8 ± 315.5/595 (350–1950)	0.200	0.119
	Mild splenomegaly	42	267.7 ± 358.2/126 (40–1290)	844.9 ± 564.3/599 (300–2315)		
	Moderate splenomegaly	11	362.4 ± 417.1/150 (50–1150)	978.3 ± 405.5/910 (619–2090)		
Lymphadenopathy	No lymphadenopathy	26	234.5 ± 296.5/99 (40–980)	685.2 ± 370.4/565 (300–1910)	0.121	0.152
	Generalized	65	294.5 ± 334.9/135 (40–1290)	839.5 ± 490.9/680 (619–2315)		
Rai stage	Low risk (I&II)	70	190.4 ± 218.4/99 (40–850)	596.2 ± 160.9/564 (300–1150)	< 0.0001	< 0.0001
	High risk (III&IV)	21	568.5 ± 438.3/280 (40–1290)	1459.3 ± 523.2/1350 (850–2315)		
WBCs (× 10^3^/µL)	< 100	60	263.2 ± 304.9/116 (40–1150)	685.2 ± 370.4/565 (300–1910)	0.728	0.541
	≥ 100	31	305.8 ± 361.7/118 (40–1290)	839.5 ± 490.9/680 (619–2315)		
Hemoglobin	Non-anemic patients	49	230.8 ± 279.8/100 (40–1200)	687.4 ± 358.5/581 (300–2090)	0.049	0.006
	Anemic patients	43	330.1 ± 363.2/140 (49–1290)	916.0 ± 514.7/780 (350–2315)		
Platelets	No thrombocytopenia	79	209.9 ± 244.1/103 (40–1150)	652.1 ± 221.9/600 (300–1580)	< 0.0001	< 0.0001
	Thrombocytopenia	12	723.4 ± 431.6/911 (206–1290)	1739.2 ± 534.4/1930 (540–2315)		
LDH (IU/L)	< 550	46	288.2 ± 329.1/104 (40–1200)	798.9 ± 491.4/632 (300–2210)	0.833	0.549
	≥ 550	45	266.9 ± 321.9/120 (40–1290)	793.9 ± 437.7/674 (350–2315)		
β2M (mg/dL)	< 3.5	35	206.3 ± 266.9/98 (40–1200)	589.3 ± 277.1/548 (300–1980)	< 0.018	< 0.0001
	≥ 3.5	56	322.3 ± 349.8/150 (46–1290)	924.3 ± 509.3/781 (400–2315)		
CD38 (%)	Negative	54	152.9 ± 191.3/95 (40–810)	576.4 ± 149.0/545 (300–910)	< 0.0001	< 0.0001
	Positive	37	460.0 ± 388.4/280 (42–1290)	1115.2 ± 571.2/910 (400–2315)		
17p del	Negative	10	180.2 ± 209.6/84 (40–687)	555.2 ± 171.3/500 (350–950)	0.002	0.003
	Positive	11	791.9 ± 435.9/950 (100–1290)	1545.2 ± 686.3/1850 (400–2315)		

Median levels of LDH and β2M were used to dichotomize into groups of low (< median) and high (≥ median)

*P1* = *p* value of CXCL-13 levels comparison among the subgroups

*P2* = *p* value of galectin-9 levels comparison among the subgroups

*WBCs* white blood counts, *LDH* lactate dehydrogenase, *β2* β2-microglobulin

### Correlation analysis of serum CXCL13 and galectin-9 with established prognostic markers

**Fig. 2:**
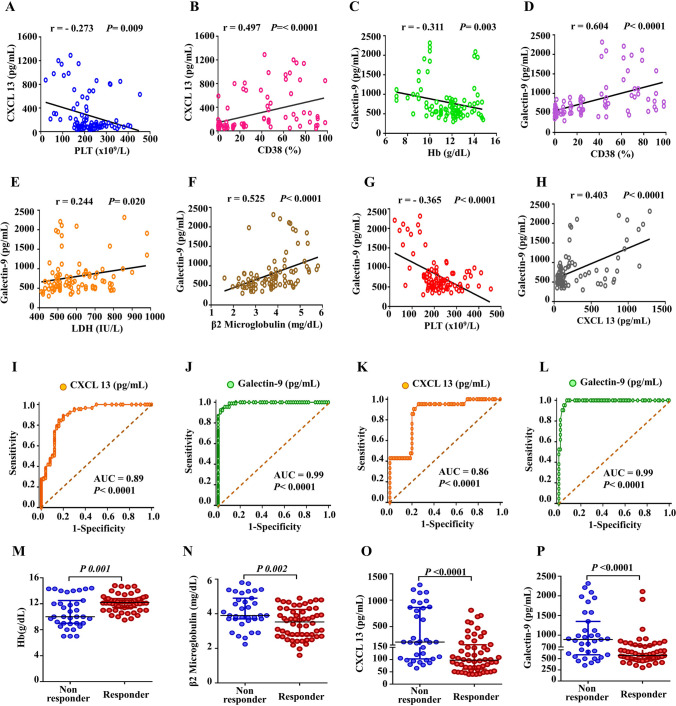
**A**–**H** Schematic box plots representing the Spearman rank correlation test of serum CXCL13 and galectin 9 levels against prognostic factors among CLL patients. First, correlation between CXCL13 and (**A**) PLT, (**B**) CD38%. Second, correlation between galectin 9 levels and (**C**) Hb, (**D**) CD38%, (**E**) LDH, (**F**) β2M, (**G**) PLT count, and (**H**) CXCL13 levels. Receiver operating characteristic (ROC) curves for discriminating cases with CLL from healthy controls by (**I**) serum CXCL13 and (**J**) galectin 9 levels. ROC curves for discriminating high- and low-risk CLL patients by (**K**) serum CXCL13 and (**L**) galectin 9 levels. Patients with the absolute cut-off value of serum CXCL13 ≥ 195 pg/mL and serum galectin 9 ≥ 860 pg/mL had a significantly higher-risk CLL. Increased Hb concentrations (**M**) and decreased serum β2M (**N**), CXCL13 (**O**) and galectin 9 (**P**) in CLL patients are predictive of future therapeutic response. Horizontal and vertical lines indicate median and interquartile range, respectively; each circle represents an individual patient sample. Hb hemoglobin, PLT platelets, LDH lactate dehydrogenase, β2M β2-microglobulin, AUC area under the curve

Correlation analysis of serum CXCL13 and galectin-9 with established prognostic markers is shown in supplementary Table S2. Statistically significant positive correlations were detected between serum galectin-9 and LDH concentrations, β2-microglobulin levels, CD38%, and serum CXCL13, but significant negative correlations were detected between serum galectin-9 and platelets numbers and hemoglobin levels. Also, a significant negative correlation was detected between CXCL13 and platelet count. Similarly, a significant negative correlation was found between hemoglobin levels and LDH concentrations (Fig. 2A–H, Figure S2). However, no associations between serum galectin-9 and CXCL13 levels and the level of bone marrow infiltration were discovered. Also, we did not find a significant correlation between serum CXCL13 and the remaining established prognostic markers (Table S2).

### Validation of the serum CXCL13 and galectin-9 in discriminating CLL patients from healthy controls and the low risk from high-risk CLL patients

Figure 2I–L and Table 2 show the ROC curve analysis results and predictive values of the serum CXCL13 and galectin-9 for the differential diagnosis of CLL patients and the controls. Serum CXCL13 and galectin-9 were considered excellent for discriminating CLL patients and the controls with cut-off values ≥ 50 pg/mL and ≥ 412 pg/mL, respectively (*p* values < 0.0001). Also, the two biomarkers combined showed higher sensitivity, specificity, positive-predictive value, and negative predictive value (97.8%, 94.0%, 96.7%, and 95.1%, respectively, with *p* value < 0.0001) for discriminating CLL patients and the controls with cut-off values ≥ 462 pg/mL. In addition, serum CXCL13 and galectin-9 were considered excellent for discriminating high-risk and low-risk CLL patients with cut-off values ≥ 195 pg/mL and ≥ 860 pg/mL, respectively (*p* values < 0.0001). Furthermore, when the two biomarkers were combined, their collaborative accuracy was excellent for discriminating high-risk and low-risk CLL patients with cut-off value ≥ 1055 pg/mL and showed a higher sensitivity, specificity, and negative predictive value (95.2%, 84.3%, and 98.3%, respectively, with *p* value < 0.0001).

**Table 2:** Receiver operating characteristic curve analysis results and predictive value of evaluated serum CXCL 13 and galectin-9 levels for the differential diagnosis of studied groups

A. ROC curve analysis results of evaluated serum CXCL 13 and galectin-9 levels for the differential diagnosis of CLL patients and healthy controls									
Variable	AUC (95%)	Cut-off	Sens (%)	Spec (%)	PPV (%)	NPV (%)	% correctly identified patients	YI	*p* value
Galectin-9 (pg/mL)	0.99 (0.98–1.00)	≥ 412	92.3%	98.0%	98.8%	87.5%	99.3%	0.903	< 0.0001
CXCL 13 (pg/mL)	0.89 (0.84–0.96)	≥ 50	89.0%	80.0%	89.0%	80.0%	89.7%	0.690	< 0.0001
Combined	0.99 (0.98–1.00)	≥ 462	97.8%	94.0%	96.7%	95.1%	96.5%	0.918	< 0.0001
B. ROC curve analysis results of evaluated serum CXCL 13 and galectin-9 levels for the differential diagnosis of high- and low-risk CLL patients									
Variable	AUC (95%)	Cut-off	Sens (%)	Spec (%)	PPV (%)	NPV (%)	% correctly identified patients	YI	*p* value
Galectin-9 (pg/mL)	0.99 (0.97–1.01)	≥ 860	95.2%	94.3%	83.3%	98.5%	94.5%	0.890	< 0.0001
CXCL 13 (pg/mL)	0.86 (0.78–0.95)	≥ 195	85.7%	80.0%	57.6%	94.9%	81.3%	0.660	< 0.0001
Combined	0.95 (0.91–0.99)	≥ 1055	95.2%	84.3%	64.5%	98.3%	86.8%	0.795	< 0.0001

*AUC* area under the curve, *Sens* sensitivity, *Spec* specificity, *PPV* positive-predictive value, *NPV* negative-predictive value, *YI* Youden’s index

The sensitivity, specificity, and positive- and negative-predictive values were calculated as follows:

• Sensitivity (Sens) = true positive/(true positive + false negative)

• Specificity (Spec) = true negative/(true negative + false positive)

• Positive-predictive value (PPV) = true positive/(true positive + false positive)

• Negative-predictive value (NPV) = true negative/(true negative + false negative)

• The percentage of correctly identified patients was (true positive + true negative)/(true positive + true negative + false positive + false negative)

• Youden’s index (YI) was calculated as (Sens + Spec) − 1

We applied comparison analysis to investigate the impact of patient clinical characteristics, serum CXCL13 and galectin-9, and other prognostic markers on CLL risk classification. Supporting our earlier findings, we discovered a statistically significant relationship between male gender, spleen size, hemoglobin levels, platelet counts, β2-microglobulin, LDH, CD38%, 17p del, serum CXCL13, serum galectin-9, and high-risk CLL (Table S3).

### Cox models with univariable and multivariate analysis of PFS in CLL patients

Age, sex, Rai stage, β2M, CD38, 17p del, galectin-9, and CXCL13 were all considered in the univariate Cox regression analysis of PFS. The results revealed that Rai stage (III&IV) (*p* = 0.006), β2M ≥ 3.5 (mg/L) (*p* = 0.006), CD38 (*p* = 0.001), positive 17p del (*p* = 0.005), galectin-9 ≥ 650 pg/mL (*p* = 0.004), and CXCL13 ≥ 120 pg/mL (*p* = 0.005) had a negative impact on PFS. The above indicators were further subjected to multivariate Cox regression analysis, which revealed that Rai stage (III&IV) (*p* = 0.001) was an independent negative prognostic variable of PFS (Table S4).

All 91 patients included in this study were treatment naïve, progressed and required treatment according to the International Workshop CLL criteria [18]. As expected, patients with high-risk CLL had poor prognosis markers and a higher percentage of disease progression and therapy resistance than low-risk patients. In support of our previous findings, we observed a significant association between the spleen size, serum CXCL13, serum galectin-9, β2-microglobulin, and hemoglobin levels at diagnosis and subsequent therapy response, demonstrating that patients with high serum CXCL13 and serum galectin-9 levels are more likely to have disease progression and therapy resistance than people with low chemokine levels (Table 3, Fig. 2M–P, and Figure S3). Hemoglobin levels, β2-microglobulin, serum CXCL13, and galectin-9 were considered excellent for predicting responders and non-responders CLL patients with cut-off values > 10 g/dL, < 3.5 mg/dL, < 120 pg/mL, and < 650 pg/mL, respectively (*p* = 0.001, *p* = 0.002, *p* < 0.0001, and *p* < 0.0001, respectively).

**Table 3:** Comparison of the responder and non-responder CLL patients

Characteristic	Category	Responder *n* = 56	Non-responder *n* = 35	*p* value
Age (years)	Mean ± SD	61.8 ± 7.1	62.6 ± 8.1	0.599
	< 65	44 (78.6%)	24 (68.6%)	0.327
	≥ 65	12 (21.4%)	11 (31.4%)	
Sex	Female	27 (48.2%)	10 (28.6%)	0.081
	Male	29 (51.8%)	25 (71.4%)	
Liver size	Normal	29 (51.8%)	17 (48.6%)	0.542
	Hepatomegaly	27 (48.2%)	14 (51.4%)	
Spleen size	Normal	32 (57.1%)	6 (17.1%)	0.007
	Splenomegaly	24 (42.9%)	29 (82.9%)	
Lymphadenopathy	No lymphadenopathy	20 (35.7%)	6 (17.1%)	0.062
	Generalized	36 (64.3%)	29 (82.9%)	
Rai stage	Low risk (I&II)	54 (96.4%)	16 (45.7%)	< 0.0001
	High risk (III&IV)	2 (3.6%)	19 (54.3%)	
WBCs (× 10^3^/µL)	Mean ± SD	100.8 ± 64.2	82.7 ± 31.2	0.519
PB lymphocytes (%)	Mean ± SD	77.2 ± 12.5	77.1 ± 15.4	0.659
Hb (g/dL)	Mean ± SD	12.1 ± 1.2	10.7 ± 2.4	0.001
Platelets (× 10^3^/µL)	Mean ± SD	221.1 ± 65.1	221.9 ± 104.5	0.604
PT (s)	Mean ± SD	13.1 ± 1.7	13.3 ± 2.2	0.574
PC%	Mean ± SD	85.2 ± 12.3	83.1 ± 15.2	0.496
LDH (IU/L)	Mean ± SD	574.8 ± 124.9	613.3 ± 141.8	0.177
β2 microglobulin (mg/dL)	Mean ± SD	3.5 ± 0.9	4.1 ± 0.9	0.001
CXCL-13 (pg/mL)	Mean ± SD	173.7 ± 187.6	444.1 ± 417.6	< 0.0001
Galectin-9 (pg/mL)	Mean ± SD	649.0 ± 311.0	1029.2 ± 565.2	< 0.0001
BM lymphocytes (%)	Mean ± SD	78.3 ± 13.4	80.2 ± 15.1	0.225
CD38 (%)	Mean ± SD	22.5 ± 29.3	46.7 ± 29.8	< 0.0001
	Negative	41 (73.2%)	13 (37.1%)	0.001
	Positive	15 (28.8%)	22 (62.9%)	
17p del	Negative	9 (81.8%)	1 (10.0%)	0.002
	Positive	2 (18.2%)	9 (90%)	

*PB* peripheral blood, *WBCs* white blood counts, *Hb* hemoglobin, *PC* prothrombin concentration, *PT* prothrombin time, *LDH* lactate dehydrogenase

### Time to first therapy and prognostic analysis of serum CXCL13 and galectin-9 concentrations in CLL patients

**Fig. 3:**
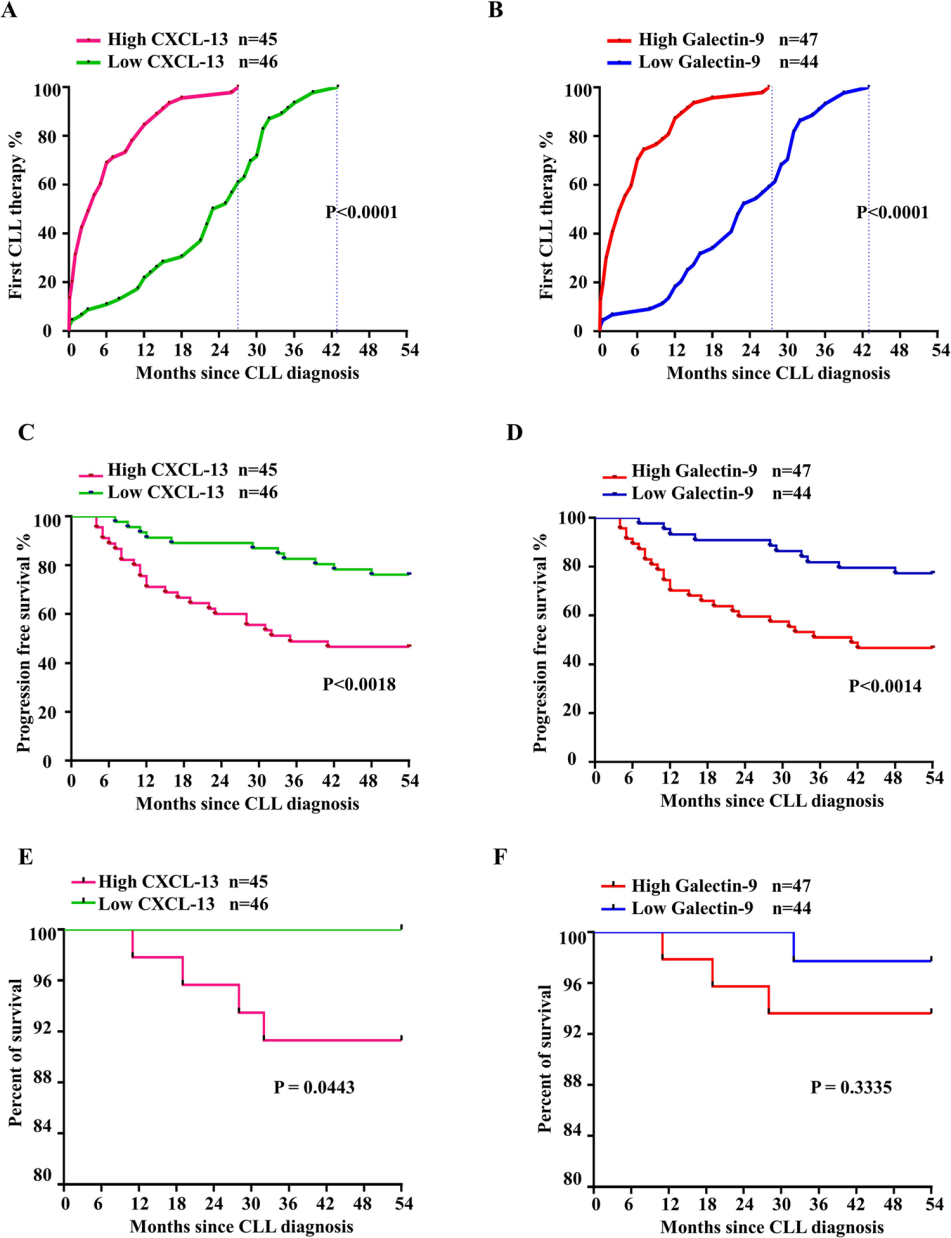
Distribution of CXCL13 and galectin 9 plasma levels and potential of time to first treatment and progression-free survival and overall survival for CLL patients’ subtypes. First, Kaplan–Meier curves for estimating the time to first therapy among newly diagnosed CLL patients based on high versus low plasma concentrations of (**A**) CXCL13 and (**B**) galectin 9. Second, Kaplan–Meier estimates progression-free survival based on high versus low plasma concentrations of (**C**) CXCL13 and (**D**) galectin 9. Third, Kaplan–Meier estimates overall survival based on high versus low plasma concentrations of (**E**) CXCL13 and (**F**) galectin 9. Sample concentrations were dichotomized into groups of patients with high or low plasma CXCL13 and galectin 9 concentrations based on the median concentration among CLL patients, i.e., CXCL13 cut-off 120 pg/mL and galectin 9 cut-off 650 pg/mL

In subsequent analysis, the group of patients with high CXCL13 levels (≥ 120 pg/mL) and low CXCL13 levels (< 120 pg/mL) was dichotomized using an approximation of the median. Also, the group of patients with high galectin-9 levels (≥ 650 pg/mL) and low galectin-9 levels (< 650 pg/mL) was dichotomized using an approximation of the median. Within CLL subgroups, there were significant differences in the median time from sample collection to first CLL treatment (Fig. 3A and B). As would be predicted, individuals with poor prognosis markers had shorter median TTFT than patients with good prognostic markers. When compared to individuals with low plasma concentrations of CXCL13, whose median TTFT was 24 months (95% CI, 0.5–43 months, *n* = 46, *p* < 0.0001), the median TTFT for CLL patients with high CXCL13 concentrations was 4 months (95% CI, 0–27 months, *n* = 45). Also, the median TTFT for CLL patients with high galectin-9 levels was 4 months (95% CI, 0–27 months, *n* = 47), which is significantly shorter than in patients with low serum galectin-9 levels (23 months, 95% CI, 0.5–43 months, *n* = 44, *p* < 0.0001).

To further understand the independent predictive relevance of serum CXCL13 and galectin-9 concentration in CLL, the Kaplan–Meier curves were used to explain the PFS and OS of CLL patients. As shown in Fig. 3C and D, patients with low levels of CXCL13 and galectin-9 were significantly associated with longer PFS in CLL (*p* = 0.0018, *p* = 0.0014, respectively). In terms of serum CXCL13 and galectin-9 levels effects on OS, we found that patients with low levels of serum CXCL13 were significantly (*p* = 0.0443) associated with longer OS, while low galectin-9 serum levels were not significantly associated with longer OS in CLL (Table S5 and Fig. 3E and F).

## Discussion

CXCL13 is one of the most essential chemokine in B-cell homing and lymph node architecture and is also involved in B-cell response and expansion [19]. A number of important in vivo studies have also emphasized the significance of the CXCL13 in the pathogenesis of CLL [20, 21]. These studies established that CLL cells move to lymph node follicles in a CXCR5-dependent manner and interact with follicular stromal cells through lymphotoxin beta receptor (LTβR), causing in CXCL13 production by the lymph node stroma, followed by CLL cell activation and proliferation [20]. It was reported that CXCL13 expression by the stromal cells rises with illness progression [21]. Ticchioni et al. also demonstrated that CXCL13 is involved in inhibiting apoptosis by inducing ERK1/2 and Akt/GSK3 pathways [22]. A research by Sivina et al. showed that CXCL13 plasma concentrations were shown to be higher in CLL patients with active advanced stage illness, larger lymph nodes, and higher CLL cell birth rates [23]. Galectin-9 is also involved in the pathogenesis of CLL by blocking the host’s anti-tumor immune responses and making changes in tumor microenvironments. According to earlier research, the CLL cells upregulate the expression of Gal-9 and PD-L1 in order to engage with the inhibitory receptors Tim-3 and PD-1, resulting in induction of exhaustion processes in infiltrating CD8 + T cells and escape of immune effector anti-tumor mechanisms [17]. Additional evidence is provided by the findings that Tim-3/Gal-9 pathway inhibition has been found to work in concert with PD-1/PD-L1 pathway inhibition to restore the function of exhausted CD8 + T cells [24, 25]. Also, Pang et al. showed that the binding of galectin-9 and Tim-3 on T regulatory cells (Treg) enhanced Treg cell function while reducing Th1 and CD4 + T cell function, which has been restored in CLL in vitro tests by blocking the galectin-9/Tim-3 pathway [26]. In a recent study, the researchers discovered that galectin-9 levels and Tim-3 was markedly elevated in CLL patients and the percentage of Tim-3 on T cells and galectin-9 levels increased as the disease progressed [12]. As a result, we could consider that CXCL13 and galectin-9 cooperate preferentially to provide a suitable tumor microenvironment by supporting the CLL cell survival, growth, proliferation, and invasion and enhancing antiapoptotic activity through CXCL13/CXCR5, galectin-9/Tim-3 pathways as well as several immunosuppressive mechanisms like enhancing the function myeloid-derived suppressor cells and exhaustion of T cell [12, 17].

By combining the assessment of the CLL disease course and the clinical merit of measuring plasma CXCL13 and galectin-9 concentrations in treatment-naive CLL patients, we found that measuring soluble CXCL13 and galectin-9 concentration could effectively determine both CLL disease activity and progression and predict treatment response. Applying this approach, we found that galectin-9 levels in CLL patients were considerably higher than that in healthy controls and increased with the progression of the Rai/Binet stage, and high-risk patients which was consistent with the findings of previous studies [12, 13]. Also, CXCL13 plasma concentrations were found to be significantly higher in CLL patients than that in healthy controls and increased with active advanced stage disease, and in those with high risk, which was in line with the prior observation [15, 16].

CD38, cytogenetic abnormalities, serum β2-MG, and serum LDH have all been reported to be prognostic indications for CLL in earlier studies [4, 6, 7]. We studied the correlation between soluble CXCL13, galectin-9, and the aforementioned markers to determine the potential prognostic importance of galectin-9 in CLL. We discovered that the groups with CD38 + , LDH > 500 IU/L, 17p del positive, and β2-MG > 3.5 mg/L had considerably higher concentrations of soluble galectin-9. Also, in the CD38 + , 17p del positive, and β2-MG > 3.5 mg/L groups, CXCL13 was considerably higher concentrations. Statistically significant positive correlations were detected between serum galectin-9 and CXCL13. Additionally, using univariate Cox analysis, it was discovered that soluble CXCL13 and galectin-9 levels were risk factors for PFS in CLL patients. According to earlier studies, they found a link between the level of galectin-9 in patients’ serum and the severity of their cutaneous T-cell lymphoma [27]. Also, solid tumors like kidney and pancreatic cancer have a poor prognosis if serum levels of galectin-9 are high [28, 29]. In line with earlier cancer research, the CXCL13 chemokine is crucial for the initiation and development of a variety of human malignancies like Waldenström macroglobulinemia and multiple myeloma [30, 31].

Furthermore, our findings of the ROC analysis demonstrated that soluble CXCL13 and soluble galectin-9 had very good specificity and sensitivity in detecting CLL disease progression. Meanwhile, using the ROC curve cut-off value, we conducted a PFS study on CXCL13 and galectin-9 and discovered that patients with high levels of CXCL13 and galectin-9 had a poor prognosis.

By directly evaluating these biomarkers before therapy and analyzing follow-up results over a long 4 years, we noticed that high initial levels of these biomarkers were associated with a higher predictive ability of short TTT and were correlated with unfavorable treatment responses, suggesting that these biomarkers may be a better predictor of CLL treatment responses.

Additional evidence could be provided by the findings that ibrutinib has been found to significantly reduce CXCL13-mediated CLL cell adherence to the BM and lymph node microenvironment in CLL patients, including interactions with macrophages [32, 33]. Also, the discovery that CXCL13 concentrations in bone marrow cell supernatants were significantly lower in CLL patients on ibrutinib [32] and elevated CXCL13 levels decrease in patients who are responding well to ibrutinib therapy and rise again in those who are developing resistance [16] provides more evidence in favor of important role of CXCL13 chemokine in CLL pathogenesis.

To the best of our knowledge, this is the first study to compare the accuracy of soluble CXCL13 and galectin-9 in assessing CLL disease progression. We showed that the prediction functions of soluble galectin-9 in determination of high-risk patients and PFS were superior to soluble CXCL13, although the two biomarkers were equal in prediction of TTT and treatment response. Meanwhile, soluble CXCL13 was superior in prediction of OS. Furthermore, collaborative accuracy of the two biomarkers together was excellent for prediction of disease activity, and discriminating high-risk CLL patients, as well as short PFS. These results could be applied to facilitate the rational design of new CLL therapies. The lack of studying treatment effects on the plasma levels of CXCL13 and galectin-9, on the other hand, is a limitation of this study that should be addressed in future research.

In conclusion, our results data indicate that the higher level of soluble galectin-9 and CXCL13 was linked to clinical progression, a worse prognosis for CLL patients, and unfavorable treatment response. Galectin-9 and CXCL13 can be combined to create more useful indicators for directing CLL clinical practice. Our study highlights the impact of galectin-9 and CXCL13 as crucial prognostic markers for CLL patients.

### Supplementary Information

Below is the link to the electronic supplementary material.

Supplementary file1 (DOCX 1130 KB)

## Data Availability

The authors confirm that all data generated or analyzed during this study are included in this article, its supplementary information files, and are available from the corresponding author on reasonable request.
