# Supplementary material for: CXC chemokine ligand 13 and galectin-9 plasma levels collaboratively provide prediction of disease activity and progression-free survival in chronic lymphocytic leukemia
Source: Ann Hematol. 2023 Nov 9;103(3):781–92. doi: 10.1007/s00277-023-05540-8 (PMC10867040; doi:10.1007/s00277-023-05540-8)
Supplement: Supplementary file 1 — Supplementary file1 (DOCX 1130 KB) [file 277_2023_5540_MOESM1_ESM.docx]

**Supplementary Appendix**

**CXC Chemokine Ligand 13 and Galectin-9 Plasma Levels Collaboratively Provide Prediction of Disease Activity and Progression-Free Survival in Chronic Lymphocytic Leukemia**

***Heba A. Ahmed ^1^, Asmaa Nafady ^2^, Eman H. Ahmed ^3^,*** ***Emad Eldin Nabil Hassan ^4^, Walaa Gamal Mohamed Soliman* ^4^, *Mahmoud I. Elbadry ^5^, Ahmed Ahmed Allam ^1^***

1 Department of Clinical Pathology, Faculty of Medicine, Sohag University, Sohag, Egypt

2 Department of Clinical and Chemical Pathology, Faculty of Medicine, South Valley University, Qena, Egypt

3 Department of Clinical Pathology, South Egypt Cancer Institute, Assiut University, Assiut, Egypt

4 Department of Clinical Oncology and Nuclear medicine, Sohag University Hospital, Sohag, Egypt.

5 Department of Internal Medicine, Division of Haematology, Faculty of Medicine, Sohag University, Sohag, Egypt.

**^++^Corresponding author:**

…………………… **^MD PhD^**

**Address:** Department of ……………………………………………,

Sohag University, Nasr City, Eastern Avenue, University Street, Sohag,

Egypt

**E-mail:**

**Postal Code:** 82524

**Phone:** +2-…………..

**Fax:** +2-093-4609304

**1 Supplementary Method**

***1.1. Methods***

**1.1.1 Flow cytometry analysis for detection of colonality of lymphocytes subsets**

Analysis of bone marrow and peripheral blood using flow cytometry was performed using CD2, CD3, CD5, CD4, CD7, CD8, CD10, CD11c, CD13, CD16, CD19, CD20, CD22, CD23,CD25, CD45, CD56, CD79b,CD123, CD200, FMC7, HLA-DR, TCR-Alpha/Beta& gamma/ delta, Kappa and Lambda. Gating using CD19 postive cells to exclude B cells (Bs) monoclonality, CD3 - T cells (Ts),CD8 - cytotoxic T cells, CD4 - helper T cells to exclude T cells monoclonality and CD16, CD56 for natural killer (NK) cells clonal evolution.

The samples of peripheral blood were taken during the study period using a sterile syringe and collected in blood collected on EDTA tube for further flow analysis. Bone marrow aspirate was stained followed by RBC lysis with ammonium chloride. Events were acquired on FACSCanto II cytometers (BDBiosciences, San Diego CA) and data were analyzed using FCSExpress (De Novo Software, Glendale CA) and the FlowJo software package, version 10.0.7 (Treestar, Ashland, OR, USA).

**1.1.2 Radiologic assessment:**

1. Computerized tomography (CT) neck, chest ,abdomine and pelvis as radiological assessment for lymphadenopathy evaluation and diasease regression or progression.
2. Abdominal ultrasound and dopplar: For optimal views of the liver, GB, kidneys, spleen, pancreas. portal vein, abdominal vessels and lymphadenopathy.
3. Echocardiography were routinely performed for screening all the patients.
4. Pan-CT/ PET-CT for follow up

**1.1.3** **Histopathological studies**

1. **Hematoxylin and Eosin staining of lymph nodes and BM biopsies.**

Hematoxylin and Eosin (H&E) staining of lymph nodes, and bone marrow, were prepared from CLL patients. The tissue sections were stained with Hematoxylin and Eosin (Hematoxylin, SR.No. AL2360, Batch No. HS502 & Eosin C.No. E30809,B.No.602023, Alpha chemika, India) as described previously.

1. **Immunohistochemistry staining**

Immunohistochemistry staining of multiples lymph nodes biopsies was prepared from some CLL patients with lymphadenopathy, using CD3, CD5, cycline D, CD15, CD19, CD20, CD30, Ki-67 for proliferation index as described previously.

**1.2 Treatment**

**Treatment Indications[**[**1**](#_ENREF_1)**]**

At least one of the following criteria should be met:

1. Evidence of progressive bone marrow failure as manifested by the development of, or worsening of, anemia and/or thrombocytopenia
2. Massive (i.e., at least 6 cm below the left costal margin) or progressive or symptomatic splenomegaly
3. Massive nodes (i.e., at least 10 cm in longest diameter) or progressive or symptomatic lymphadenopathy
4. Progressive lymphocytosis with an increase of more than 50% over a 2-month period or lymphocyte doubling time (LDT) of less than 6 months after exclusion of infections.
5. Autoimmune anemia and/or thrombocytopenia that is poorly responsive to corticosteroids or other standard therapy
6. Constitutional symptoms, defined as any one or more of the following disease-related symptoms or signs: a. Unintentional weight loss of 10% or more within the previous 6 months; b. significant fatigue (i.e., ECOG PS 2 or worse; inability to work or perform usual activities); c. fevers higher than 100.5°F or 38.0°C for 2 or more weeks without other evidence of infection; or d. night sweats for more than 1 month without evidence of infection.
7. Extranodal: Symptomatic or functional extranodal involvement (e.g., skin, kidney, lung, spine)

Many treatment modalities in addition to supportive measurements, were given to patients as follows….

- Cyclophosphamide, vincristine, and prednisone (CVP), or fludarabine, cyclophosphamide ± rituximab (FCR), or Bruton tyrosine kinases inhibitors (BTKIs) were used as first lines therapy depending on age, comorbidity, and del17p13.
- 21/91 patients were receiving treatment by Ibrutinib, 13 patients as first line therapy and 8 patients as seconde line therapy. Three patients discontinued ibrutinib completely due to heart problems adverse event (2 cases with atrial fibrillation and one case with mutifocal ventricular ectopics and one case with non ST elevation myocardial infarction.

**1.3 Outcomes definitions**

Response Criteria [[1](#_ENREF_1)]

| **Parameter** | **CR** | **PR** | **PD** |
| --- | --- | --- | --- |
| **Group I (**criteria define the tumor load**)** | | | |
| Lymphadenopathy* | None > 1.5 cm | Decrease ≥50% | Increase ≥50% |
| Hepatomegaly | None | Decrease ≥50% | Increase ≥50% |
| Splenomegaly | None | Decrease ≥50% | Increase ≥50% |
| Blood lymphocytes | < 4000/µL | Decrease ≥50%  from baseline | Increase ≥50% over  baseline# |
| Marrow | Normocellular, <30% lymphocytes, no B-lymphoid nodules.  Hypocellular marrow defines CRi | 50% reduction in  marrow infiltrate, or B-lymphoid nodules | Not applicable |
| **Group II** (criteria define the function of the hematopoietic system [or marrow]). | | | |
| Platelet count | > 100,000/µL | > 100,000/µL or  increase ≥50% over baseline | Decrease of ≥50%  from baseline secondary to CLL |
| Hemoglobin | > 11 g/dL | > 11g/dL or increase ≥50% over  baseline | Decrease of >2 g/dL from baseline  secondary to CLL |
| Neutrophils | > 1500/µL | > 1500/µL or >50%  improvement over baseline | Not applicable |

- **CR (complete remission):** all of the criteria have to be met, and patients have to lack disease- related constitutional symptoms
- **CRi:** CR with incomplete hematopoietic recovery
- **PR (partial remission):** at least two of the criteria of group I plus one of the criteria of group II have to be met
- **SD (statinary disease)** is absence of progressive disease (PD) and failure to achieve at least a PR
- **PD (progressive disease):** at least one of the above criteria of group I or group II has to be met.
- *Sum of the products of multiple lymph nodes (as evaluated by CT scans in clinical trials).

**Supplemental Results**

**Table S1. General data of CLL group and healthy controls group.**

|  | **CLL n=91** | **Healthy controls n=50** | **P-value** |
| --- | --- | --- | --- |
| **Sex: Male n(%)**  **Female n(%)** | 54(59.3%)  37(40.7%) | 29(58.0%)  21(42.0%) | 1.00 |
| **Age (years)** | 62.1±7.3 | 60.2±8.5 | 0.349 |
| **WBCs(x 10^3^/uL) ,** | 93.8±54.5 | 7.7±2.8 | **<0.0001*** |
| **Lymphocytes %** | 77.2±13.6 | 31.2±12.9 | **<0.0001*** |
| **Hb (g/dL)** | 11.6±1.9 | 14.0±9.6 | **<0.0001*** |
| **Platelets (x 10^3^/uL)** | 217.5±82.1 | 279.1±84.8 | **<0.0001*** |
| **CXCL13 (pg/ml)** | 277.7±323.9 | 55.9±48.1 | **<0.0001*** |
| **Galectin-9 (pg/ml)** | 759.4±463.0 | 272.1±60.5 | **<0.0001*** |

**Table S2. Correlations** **of CXCL 13 and** **Galectin-9 levels with prognostic factors in 91 CLL patients.**

|  | **Galectin-9 (pg/ml)** | | **CXCL13 (pg/ml)** | |
| --- | --- | --- | --- | --- |
|  | **r** | **P-value** | **r** | **P-value** |
| **CXCL-13 (pg/ml)** | 0.403 | <0.0001* |  |  |
| **Age (years)** | -0.075 | 0.479 | -0.082 | 0.441 |
| **WBCs(x 10^3^/uL) ,** | -0.002 | 0.984 | 0.056 | 0.598 |
| **Hb (g/dL)** | -0.311 | 0.003* | -0.135 | 0.201 |
| **LDH (IU/L)** | 0.244 | 0.020* | 0.164 | 0.121 |
| **β2 Microglobulin (mg/dL)** | 0.525 | <0.0001* | 0.117 | 0.268 |
| **Platelets (x 10^3^/uL)** | -0.365 | <0.0001* | -0.273 | 0.009* |
| **CD38 (%)** | 0.604 | <0.0001* | 0.497 | <0.0001* |
| **PT (sec)** | 0.194 | 0.065 | 0.207 | 0.049* |
| **PC (%)** | -0.204 | 0.052 | -0.186 | 0.077 |
| **CD79b(%)** | -0.002 | 0.984 | 0.177 | 0.094 |
| **CD22(%)** | 0.088 | 0.406 | 0.208 | 0.047* |
| **CD5(%)** | 0.166 | 0.116 | 0.189 | 0.073 |
| **CD19(%)** | -0.108 | 0.309 | -0.096 | 0.366 |
| **CD 45(%)** | 0.049 | 0.642 | -0.077 | 0.466 |
| **Sigm(%)** | -0.078 | 0.465 | -0.103 | 0.330 |
| **Kappa (%)** | 0.209 | 0.047* | 0.151 | 0.153 |
| **Lambda (%)** | -0.088 | 0.406 | -0.046 | 0.664 |
| **CD20(%)** | -0.019 | 0.860 | -0.045 | 0.673 |
| **FMC7(%)** | 0.164 | 0.120 | 0.096 | 0.366 |
| **CD23(%)** | 0.088 | 0.408 | 0.022 | 0.839 |
| **PB lymphocytes (%)** | -0.045 | 0.670 | 0.037 | 0.730 |
| **BM lymphocytes (%)** | 0.024 | 0.825 | 0.060 | 0.575 |
| **CD 200(%)** | -0.046 | 0.664 | -0.062 | 0.556 |

Abbreviation: PB; peripheral blood, WBCs; white blood counts , Hb; hemoglobin, , PC; prothrombin concentration, PT; prothrombin time, LDH; lactate dehydrogenase.

**Table S3. Comparison of the low and high risk CLL patients.**

| **Characteristic** | **Category** | | **All CLL cases**  **n=91** | **Low risk**  **(Rai stage I&II )**  **n=70** | **High**  **(Rai stage III& IV)**  **n=21** | **P value** |
| --- | --- | --- | --- | --- | --- | --- |
| **Age (years)** | **Mean ±SD** | | 62.1 ± 7.3 | 62.4 ± 7.6 | 61 ± 6.9 | 0.521 |
| **Sex** | **Female** | | 37(40.7%) | 35(50.0%) | 2(9.5%) | **0.001** |
|  | **Male** | | 54(59.3%) | 35(50.0%) | 19(90.5%) |  |
| **Liver size** | **Normal** | | 46(50.5%) | 34(48.6%) | 12(57.1%) | 0.780 |
|  | **Mild hepatomegaly** | | 31(34.1%) | 25(35.7%) | 6(28.6%) |  |
|  | **Moderate hepatomegaly** | | 14(15.4%) | 11(15.7%) | 3(14.3%) |  |
| **Spleen size** | **Normal** | | 38(41.8%) | 33(47.1%) | 5(23.8%) | **0.002** |
|  | **Mild splenomegaly** | | 42(47.1%) | 33(47.1%) | 9(42.9%) |  |
|  | **Moderate splenomegaly** | | 11(12.1%) | 4 (5.7%) | 7(33.3%) |  |
| **Lymphadenopathy** | **No lymphadenopathy** | | 26(28.6%) | 23(32.9%) | 3 (14.3%) | 0.080 |
|  | **Generalized** | | 65(71.45) | 47(67.1%) | 18(85.7%) |  |
| **WBCs(x 10^3^/uL) ,** | **Mean ±SD** | | 93.9 ± 54.5 | 87.2 ± 37.2 | 95.9 ± 58.7 | 0.994 |
| **PB lymphocytes (%)** | **Mean ±SD** | | 77.1 ± 13.6 | 76.6 ± 13.5 | 79.1 ± 13.9 | 0.388 |
| **Hb (g/dL)** | **Mean ±SD** | | 11.6 ± 1.9 | 12.1 ± 1.3 | 9.8 ± 2.4 | **<0.0001** |
| **Platelets (x 10^3^/uL)** | **Mean ±SD** | | 217.5 ± 82.1 | 234.8 ± 68.8 | 159.8 ± 97.2 | **<0.0001** |
| **PT (sec)** | **Mean ±SD** | | 13.1 ± 1.9 | 13.1 ± 1.7 | 13.6 ± 2.3 | 0.280 |
| **PC%** | **Mean ±SD** | | 84.4 ± 13.5 | 85.3 ± 12.8 | 81.4 ± 15.9 | 0.257 |
| **LDH (IU/L)** | **Mean ±SD** | | 589.6 ± 132.2 | 565.9 ± 111.4 | 668.3 ± 165.3 | **0.005** |
| **β2 Microglobulin (mg/dL)** | **Mean ±SD** | | 3.6 ± 0.9 | 3.4 ± 0.8 | 4.7 ± 0.8 | **<0.0001** |
| **CXCL-13 (pg/ml)** | **Mean ±SD** | | 277.9 ± 323.9 | 190.4 ± 218.4 | 568.5 ± 438.3 | **<0.0001** |
| **Galectin-9 (pg/ml)** | **Mean ±SD** | | 795.5 ± 463.0 | 596.2 ± 160.9 | 1459.3 ± 523.2 | **<0.0001** |
| **Bone marrow cellularity** | **Normocellular** | | 18(19.8%) | 16(22.9%) | 2(9.5%) | 0.150 |
|  | **Hypercellular** | | 73(80.2%) | 54(77.1%) | 19(90.5%) |  |
| **BM lymphocytes (%)** | **Mean ±SD** | | 79.0 ± 13.7 | 77.9 ± 13.4 | 82.9 ± 14.3 | 0.043 |
| **CD5 (%)** | **Mean ±SD** | | 60.6 ± 21.5 | 58.4 ± 21.9 | 68.1 ± 18.9 | 0.066 |
| **CD19 (%)** | **Mean ±SD** | | 78.9 ± 18.8 | 79.9 ± 17.7 | 75.2 ± 22.4 | 0.614 |
| **CD20 (%)** | **Mean ±SD** | | 45.8 ± 16.9 | 44.7 ± 16.2 | 49.4 ± 19.5 | 0.212 |
| **CD22 (%)** | **Mean ±SD** | | 15.3 ± 23.1 | 14.5 ± 22.0 | 17.9 ± 26.9 | 0.741 |
| **CD23 (%)** | **Mean ±SD** | | 46.6 ± 24.7 | 45.9 ± 23.4 | 48.8 ± 29.4 | 0.713 |
| **CD38 (%)** | **Mean ±SD** | | 31.8 ± 31.6 | 21.4 ± 27.6 | 66.5 ± 15.3 | **<0.0001** |
| **CD79b (%)** | **Mean ±SD** | | 18.8 ± 18.7 | 19.9 ± 19.9 | 15.4 ± 14.2 | 0.329 |
| **FMC7 (%)** | **Mean ±SD** | | 22.8 ± 26.9 | 21.5 ± 25.2 | 27.3 ± 32.5 | 0.810 |
| **Surface immunoglobulin (%)** | **Mean ±SD** | | 32.4 ± 18.1 | 32.5 ± 19.4 | 31.9 ± 13.5 | 0.742 |
| **Kappa (%)** | **Mean ±SD** | | 52.5 ± 32.8 | 48.0 ± 33.3 | 67.2 ± 26.8 | **0.033** |
| **Lambda (%)** | **Mean ±SD** | | 21.2 ± 22.1 | 22.8 ± 23.9 | 15.6 ± 12.9 | 0.759 |
| **CD200 (%)** | **Mean ±SD** | | 46.6 ± 15.5 | 45.8 ± 14.1 | 49.4 ± 19.5 | 0.248 |
| **17p del** | **Negative** | | 10(47.6%) | 9(75.0%) | 8 (88.9%) | **0.008** |
|  | **Positive** | | 11(52.4%) | 3(25.0%) | 1(11.1%) |  |
| **Treatment response** | **Responder** | **Complete remission** | 32(35.2%) | 32(45.7%) | 0(0.0%) | **<0.0001** |
|  |  | **Partial remission** | 24(26.4%) | 22(31.4%) | 2(9.5%) |  |
|  | **Non responder** | **Progression** | 13(14.4%) | 7(10.0%) | 6(28.6%) |  |
|  |  | **Refractory** | 22(24.2%) | 9(12.9%) | 13(61.9%) |  |
| **Prognosis** | **Survival** | | 87(95.6%) | 69(98.6%) | 18(75.3%) | **0.037** |
|  | **Non-Survival** | | 4(4.4%) | 1(1.4%) | 3(14.3%) |  |

Abbreviation: PB; peripheral blood, WBCs; white blood counts , Hb; hemoglobin, , PC; prothrombin concentration, PT; prothrombin time, LDH; lactate dehydrogenase.

**Table S4. Univariable and multivariate Cox regression analysis of PFS in CLL patients**

| **Characteristic** | **Univariate analysis**  **HR (95% CI)** | **P value** | **Multivariate analysis**  **HR (95% CI)** | **P value** |
| --- | --- | --- | --- | --- |
| **Age (years) ≥65** | 1.68 (0.65–4.38) | 0.29 | - | - |
| **Male** | 2.32 (0.95–5.73) | 0.066 | - | - |
| **Rai Stage (III&IV )** | 10.0 (1.91–52.29) | **0.006** | 32.6(3.32-24.57) | 0.001 |
| **β2 Microglobulin ≥ 3.5 mg/dL** | 4.0 (1.51–10.66) | **0.006** | 1.72(0.55-5.34) | 0.352 |
| **CD38 ≥30%** | 4.6 (1.87–11.44) | **0.001** | 1.54(0.32-7.39) | 0.589 |
| **17p del positive** | 2.5 (0.20–3.23) | **0.005** | 0.04(0.01-1.19) | 0.063 |
| **CXCL-13 ≥120 pg/ml** | 3.6 (1.48–8.90) | **0.005** | 1.71(0.34-4.02) | 0.802 |
| **Galectin-9 ≥650 pg/ml** | 3.9 (1.56–9.58) | **0.004** | 1.03(0.30-3.52) | 0.960 |

**Table S5. Progression-free survival and overall survival for CLL patients subtypes.**

|  | **Group** | **Number of patients** | **Number of events** | **Median months** | **95% CI** | **P value** |
| --- | --- | --- | --- | --- | --- | --- |
| Progression free survival | CXCL-13 (+) ≥120  CXCL-13 (-) <120 | 45  46 | 24  11 | 35  54 | 1.503 - 5.74  0.174 - 0.665 | **0.0018** |
|  | Galectin 9 (+) ≥650  Galectin 9 (-) <650 | 47  44 | 25  10 | 41  54 | 1.536 - 5.82  0.172 - 0.651 | **0.0014** |
| Overall survival | CXCL-13 (+) ≥120  CXCL-13 (-) <120 | 45  46 | 4  0 | 54  54 | 1.053 - 53.09  0.018 - 0.945 | **0.0443** |
|  | Galectin 9 (+) ≥650  Galectin 9 (-) <650 | 47  44 | 3  1 | 54  54 | 0.370 - 18.68  0.053 - 2.70 | 0.3254 |

-Median levels of CXCL-13, and Galectin 9, were used to dichotomize into groups of low (<median) and high (≥median).

**Figure S1**.**17p (p53) by FISH.** Representative images of FISH analysis of CLL patient with positive 17p (p53) deletion. The patient has 17p (p53) deletion in 18% of the studies interphase nuclei (36/200). **A** Normal 17p13 copy numbers as indicated by two red 17p13 signals and **B**. heterozygous deletion as indicated by the lack of one green 17p13 signal


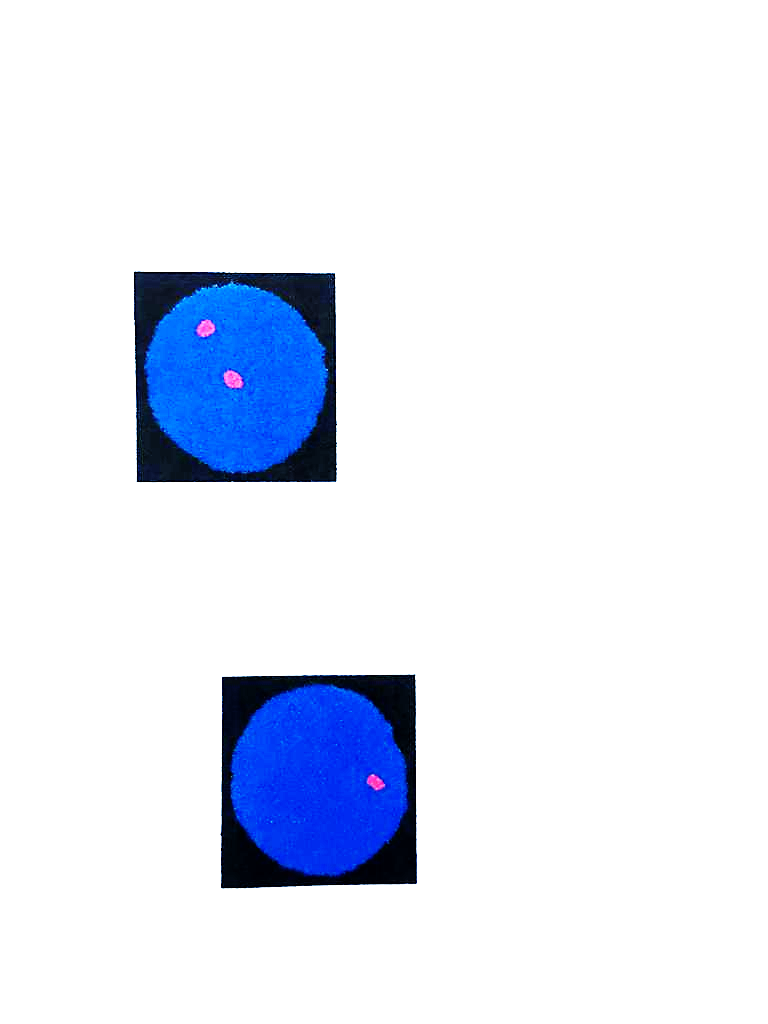


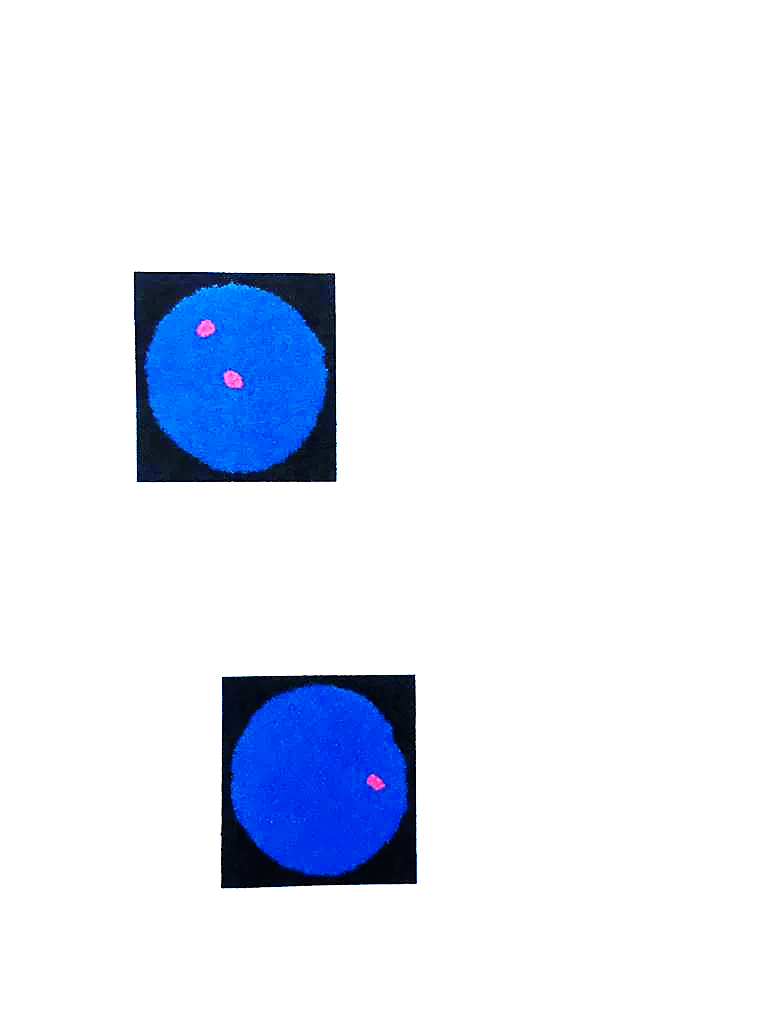
**A B**

**Figure S2.**Schematic box plots representing the Spearman rank correlation test of Hb and levels of LDH and β2M among CLL patients.

**
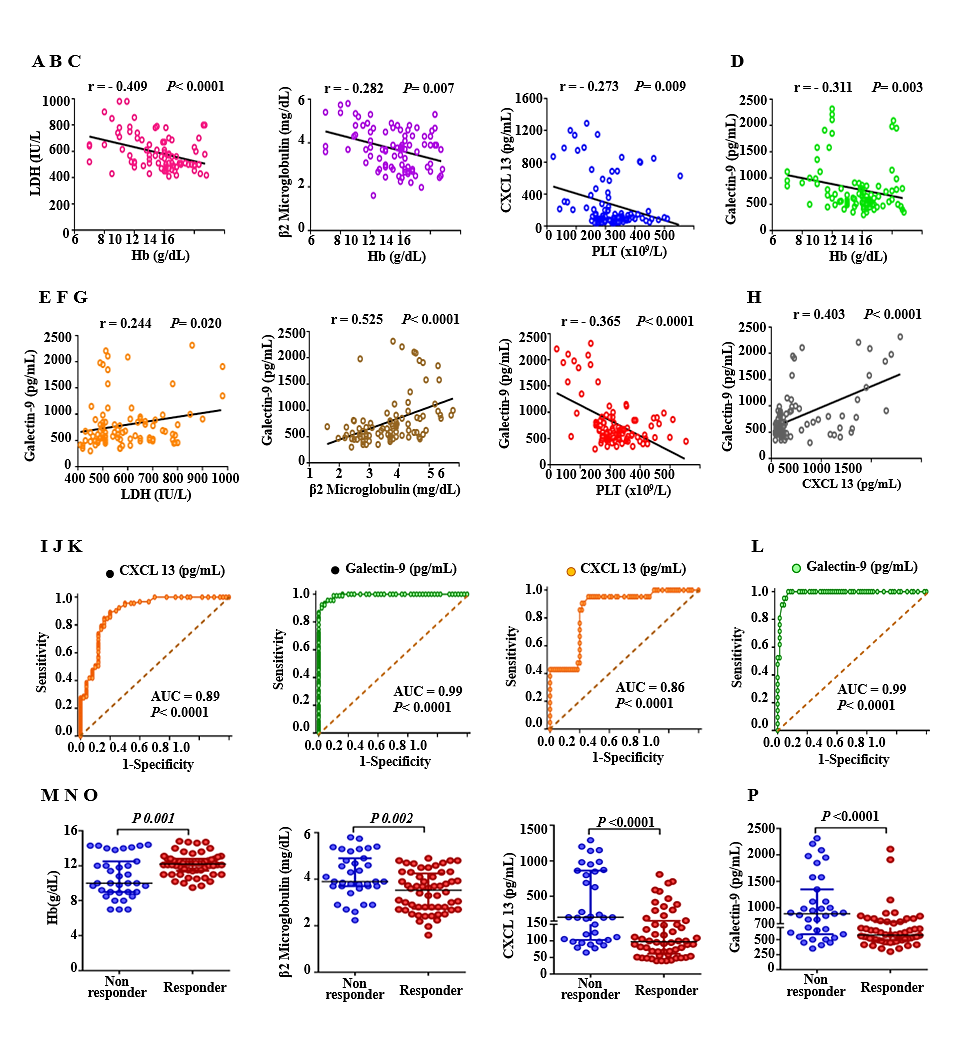
**

**Figure S3**. ROC curve analysis results of evaluated serum Hb, β2 Microglobulin , CXCL 13 and Galectin-9 levels for the differential treatment responder and non-responder CLL patients


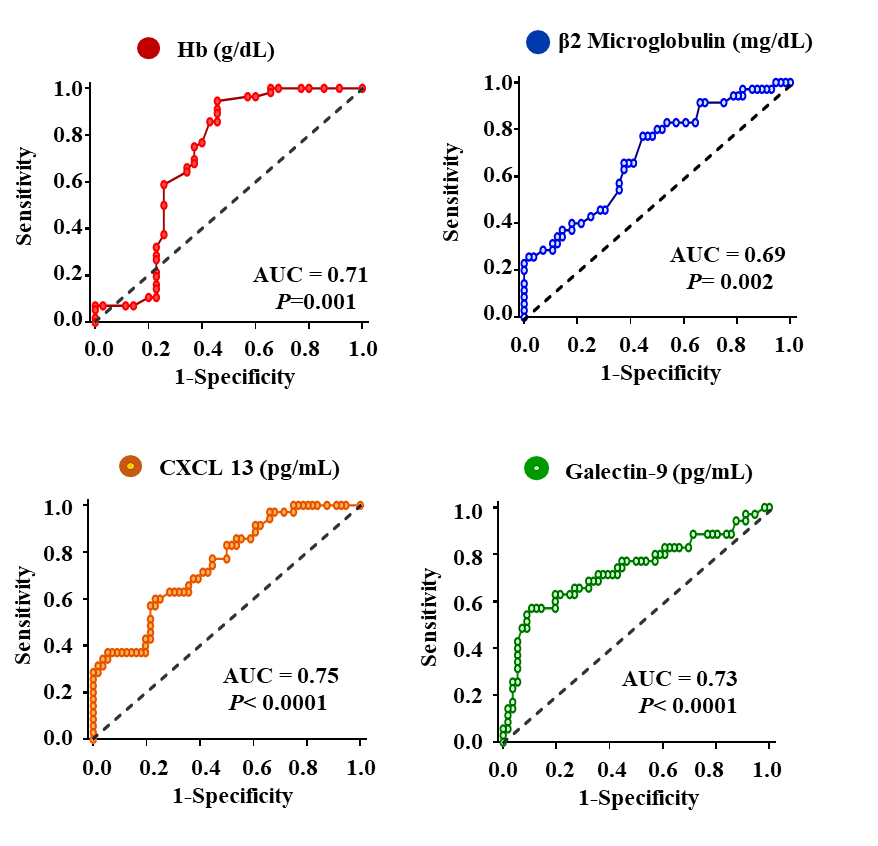


| **Variable** | **AUC (95%)** | **Cut-off** | **Sensitivity(%)** | **Specificity(%)** | ***P***  **Value** |
| --- | --- | --- | --- | --- | --- |
| **Hb (g/dL)** | 0.71(0.58-0.83) | >10.5 | 89.3% | 54.3% | **0.001** |
| **β2 Microglobulin (mg/dL)** | 0.89(0.58-0.80) | <3.5 | 80.0% | 50.0% | **0.002** |
| **Galectin-9 (pg/mL)** | 0.73(0.62-85) | <650 | 71.4% | 60.8% | **<0.0001** |
| **CXCL 13 (pg/mL)** | 0.75(0.64-0.85) | <120 | 68.6% | 64.3% | **<0.0001** |

**References**

1. Hallek M, Cheson BD, Catovsky D, Caligaris-Cappio F, Dighiero G, Döhner H, Hillmen P, Keating MJ, Montserrat E, Rai KR: **Guidelines for the diagnosis and treatment of chronic lymphocytic leukemia: a report from the International Workshop on Chronic Lymphocytic Leukemia updating the National Cancer Institute–Working Group 1996 guidelines**. *Blood, The Journal of the American Society of Hematology* 2008, **111**(12):5446-5456.
